# Clinical Outcomes from Unselected “Real-World” Patients with Long Coronary Lesion Receiving 40 mm Biodegradable Polymer Coated Sirolimus-Eluting Stent

**DOI:** 10.1155/2015/613089

**Published:** 2015-10-22

**Authors:** Anurag Polavarapu, Raghava Sarma Polavarapu, Jayesh Prajapati, Kamlesh Thakkar, Asif Raheem, Tamanpreet Mayall, Ashok Thakkar

**Affiliations:** ^1^Lalitha Super Specialty Hospital Pvt. Ltd., Heart and Brain Centre, Kothapet, Guntur, Andhra Pradesh 522001, India; ^2^Apollo Hospitals, Ahmedabad, Gujarat 382428, India; ^3^Lions Sterling Super Specialty Hospital, Mehsana, Gujarat 384002, India; ^4^Yashfeen Cardiac Hospital, Navsari, Gujarat 396445, India; ^5^Department of Clinical Trials, Sahajanand Medical Technologies Pvt. Ltd., Surat, Gujarat 395004, India

## Abstract

*Background*. Long lesions being implanted with drug-eluting stents (DES) are associated with relatively high restenosis rates and higher incidences of adverse events. *Objectives*. We aimed to examine the safety and efficacy of the long (40 mm) biodegradable polymer coated Indolimus sirolimus-eluting stent (SES) in real-world patients with long coronary lesions. *Methods*. This study was observational, nonrandomized, retrospective, and carried out in real-world patients. A total of 258 patients were enrolled for the treatment of long coronary lesions, with 40 mm Indolimus. The primary endpoints in the study were incidence of major adverse cardiac events (MACE), a miscellany of cardiac death, myocardial infarction (MI), target lesion revascularization (TLR) or target vessel revascularization (TVR), and stent thrombosis (ST) up to 6-month follow-up. *Results*. The study population included higher proportion of males (74.4%) and average age was 53.2 ± 11.0 years. A total of 278 lesions were intervened successfully with 280 stents. The observed MACE at 6-month follow-up was 2.0%, which included 0.8% cardiac death and 1.2% MI. There were no TLR or TVR and ST observed during 6-month follow-up. *Conclusions*. The long (40 mm) Indolimus stent demonstrated low MACE rate and was proven to be safe and effective treatment for long lesions in “real-world” patients.

## 1. Introduction

In the recent era, stent implantation has become the treatment of choice among patients with coronary artery disease [1–3]. Reestablishment of coronary blood flow with subsequent relief of symptoms can be successfully established in most patients. Depending on various mystifying factors, such as the presence or absence of diabetes mellitus, the size of the targeted coronary artery, the length of the coronary lesion, and the degree of vessel patency achieved by the intervention, restenosis at the site of stent implantation is seen in 15–60% of patients [4–6].

Approximately 20% of patients with coronary artery disease suffer from long lesion diseases [7, 8]. Longer stented lesion intervened with bare metal stents is an independent predictor of restenosis and adverse events [9]. Long stenting is usually associated with prolonged intracoronary modification due to multiple and overlapping stent placement, which may lead to injury to the vessel wall integrity [10]. However, despite the strong antirestenotic efficacy of drug-eluting stents (DES), the benefits of DES are often attenuated in patients with long coronary artery lesions, which are associated with an additional risk of adverse clinical outcomes [11]. Furthermore, the earlier DES with durable polymer were associated with delayed arterial healing and inflammation, which had potential tendency for late thrombosis, especially in high-risk lesions such as long coronary segments [8, 12].

The Indolimus (Sahajanand Medical Technologies Pvt. Ltd., Surat, India) employs 60 *μ*m thin-strut, L605 cobalt chromium alloy as its stent platform. The metal stent is coated with biodegradable polymer that releases sirolimus over a period of time. The biodegradable polymer undergoes degradation in 9–12 months leaving only a metal stent along the arterial wall. Though there are less reported adverse events and restenotic rates on using biodegradable polymer based DES, there are still wrangles on safety and efficacy of long DES. We therefore conducted this retrospective, observational, multicenter, nonrandomized study to examine the safety and efficacy of the long (40 mm), Indolimus sirolimus-eluting stent (SES) in real-world patients with long coronary lesions.

## 2. Methods

### 2.1. Study Design and Patient Population

This study was observational, nonrandomized, retrospective, and carried out in real-world patients at four different clinical centres of India. A total of 258 patients were enrolled for the treatment of long coronary lesions, with 40 mm Indolimus SES (Sahajanand Medical Technologies Pvt. Ltd, India). The study was held between April 2012 and June 2014. The study was conducted in accordance with the Declaration of Helsinki and country-specific regulatory requirements. All patients signed informed consent form which was reviewed and approved by the Institutional Review Board or Independent Ethics Committee.

The inclusion criteria for the study were as follows: (1) patients of age 18 years or above, (2) patients who had stable or unstable angina or acute recent myocardial infarction, and (3) patients who were undergoing coronary intervention with the long (40 mm) study stent. The patients were excluded if they refused to give written informed consent or if they had any allergy to aspirin, clopidogrel, ticlopidine, heparin, cobalt chromium, sirolimus, or polymers used in a study stent.

### 2.2. Description of the Study Stent

**Figure 1:**
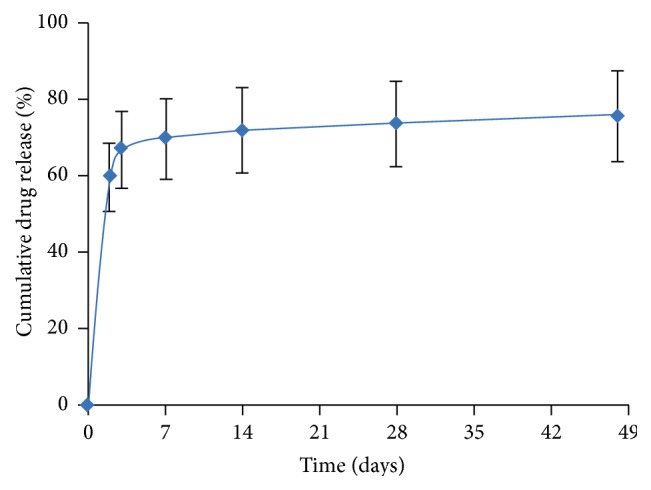
In vitro drug release from Indolimus stent.

The Indolimus biodegradable polymer coated sirolimus-eluting coronary stent (Sahajanand Medical Technologies Pvt. Ltd., Surat, India) involves L605 cobalt chromium (Co-Cr) alloy as its stent platform. The biodegradable polymer gives it a strut thickness of 60 *μ*m and drug load of 1.4 *μ*g/mm^2^. About 70% of drug is released within 7 days and remaining drug is released over a period of 48 days (Figure 1). The drug is released within 7 weeks after the stent implantation from the polymeric layers coated onto the surface of the stent. The biodegradable polymeric film is a blend of different biodegradable polymers, poly(L-lactide), 50/50 poly(D,L-lactide-co-glycolide), and polyvinylpyrrolidone, which undergoes hydrolysis. This process takes approximately 9 to 12 months after which all the polymers degrade naturally and excrete from body in the form of their metabolites.

The average coating thickness of Indolimus stent is between 5 and 6 *μ*m. The Indolimus stent is available in lengths of 8, 12, 16, 20, 24, 28, 32, 36, and 40 mm and available diameters were 2.5, 2.75, 3.0, and 3.5 mm.

### 2.3. Interventional Procedure and Adjunctive Medication

In each patient, initially access to either femoral artery or radial artery or brachial artery was created by a device called an introducer needle. Once access to the artery was gained, a sheath introducer was positioned in the opening to keep the artery open and control bleeding. Through this sheath, a long plastic tube, that is, guiding catheter, was pushed. The tip of guiding catheter was located at the opening of the coronary artery. Radioopaque dyes were injected through the guiding catheter into the coronary artery, so that the disease state and location were readily assessed by coronary angiography. During angiography, cardiologist anticipated the size of the coronary artery and decided the type of balloon catheter and coronary guidewire that would be used during intervention. The coronary guidewire was inserted through the guiding catheter into the coronary artery. The cardiologist guided the wire through the coronary artery to the site of the stenosis. The tip of the wire was then passed across the blockage. The tip of the balloon catheter was hollow and was then inserted at the back of the guidewire; thus the guidewire is now inside of the balloon catheter. The balloon catheter was tenderly pushed forward, until the deflated balloon was inside of the blockage. The balloon was then inflated, and it trampled the atheromatous plaque and stretched the artery wall to expand. The Indolimus stent on the balloon also expanded and thus implanted onto the culprit vessel with long lesion.

All patients received a loading dose of 300 mg of aspirin and clopidogrel (300 mg) or prasugrel (60 mg) or ticagrelor (90 mg). The procedural anticoagulation was brought about either with heparin or bivalirudin. However, the intraprocedural administration of glycoprotein IIb/IIIa-inhibitor was at the investigator's volition. The procedure was performed according to the standard treatment guidelines of each participating centre. All the patients received dual antiplatelet therapy (aspirin 75–300 mg/day indefinitely and clopidogrel 75 mg/day or prasugrel 10 mg/day or ticagrelor 90 mg twice daily for at least 6 months) after the procedure.

### 2.4. Endpoints of the Study

The primary endpoint of the study was major adverse cardiac events (MACE), which is a miscellany of cardiac death, myocardial infarction (MI) (Q-wave and non-Q-wave) and target lesion revascularization (TLR), target vessel revascularization (TVR), and stent thrombosis (ST). These endpoints were observed during in-hospital stay and at 30-day and 6-month follow-up. MACE will be evaluated as secondary endpoints at 12 and 24 months.

### 2.5. Definition of Endpoints and Clinical Events

Procedural success was defined in terms of in-hospital MACE. MACE is composed of cardiac death, MI, TLR, and TVR. Death can be cardiac or noncardiac death. Any death due to undetermined cause was reported as cardiac death. Q-wave MI was considered when there was development of new Q-wave of more than 0.04 seconds in two or more adjoining leads along with increase in cardiac markers like Troponin I or T, creatine kinase, or MB isoform. Non Q-wave MI was considered when there was more than three times' elevation in creatinine kinase levels along with elevation in MB isoform and Troponin marker T or I without development of new Q-waves [13]. TLR was considered when there was stenosis in treated segment (5 mm proximal and 5 mm distal edges). TVR was considered when there was stenosis in any segment of the treated vessel. ST was considered acute when it occurred within 24 hours, subacute when it occurred between 1 and 30 days, and late when it occurred after 30 days [13]. “Definite” ST was defined by symptoms suggestive of an acute coronary syndrome and angiographic or pathologic confirmation of stent thrombosis. “Probable” ST was described as unexplained death within 30 days or target vessel myocardial infarction without angiographic confirmation of stent thrombosis. “Possible” ST was defined as any unexplained death after 30 days [14].

### 2.6. Follow-Up

All patients underwent 30-day and 6-month follow-up. Clinical follow-up was scheduled by telephone communication or clinic visit at 30 days and 6 months. Follow-up data were collected pertaining to current clinical status, prior hospitalisation, and occurrence of any of the aforementioned adverse events. Telephonic follow-up was done in 160 (62.02%) patients and remaining 98 (37.98%) patients underwent clinical visit at 30-day follow-up. At 6 months, 148 (57.81%) patients were followed up telephonically and the other 108 (42.19%) patients underwent clinical follow-up. Further follow-up is scheduled to be taken at 12 months and at 24 months.

### 2.7. Statistical Analysis

Continuous variables are presented as mean ± standard deviation and categorical variables as counts and percentages. The event-free survival curve was calculated according to the Kaplan-Meier method. All data were analysed using the Statistical Package for Social Sciences (SPSS; Chicago, IL, USA) program, version 15.

## 3. Results

### 3.1. Baseline Demographics and Lesion Characteristics

A total of 258 patients were enrolled in the study. The basic demographic details of the patients are outlined in Table 1. The study population included higher proportion of males (*n* = 192; 74.4%) and average age was 53.2 ± 11.0 years. The rates of hypertension and diabetes were more common in the enrolled patients, 99 (38.4%) and 83 (32.2%), respectively. Sixty-six (25.6%) patients suffered with unstable angina. A total of 278 lesions were intervened successfully with 280 stents (1.0 ± 0.1 per lesion). The average stent length and diameter were 40.0 ± 0.0 mm and 3.1 ± 0.4 mm, respectively. A considerable number of patients had a history of previous PCI (15.5%) and 10 (3.9%) patients had a family history of CAD. Type C lesions accounted for 278 (100%) and there were 61 (21.9%) patients with chronic total occluded lesions. The lesion and angiographic procedural details are outlined in Table 2.

### 3.2. Clinical Outcomes

**Figure 2:**
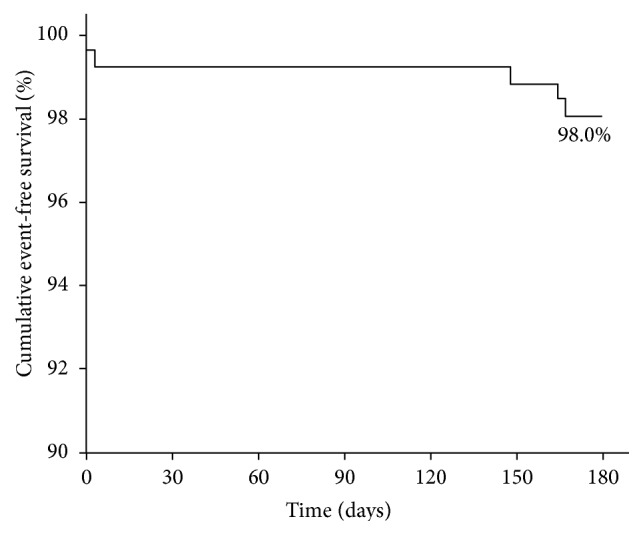
Cumulative event-free survival curve at 6-month follow-up.

Clinical follow-up was completed in 256 (99.2%) patients at the end of 6 months. The incidence of MACE during in-hospital stay and at 30 days and 6 months was found to be 2 (0.8%), 2 (0.8%), and 5 (2.0%), respectively. The detailed clinical outcomes of the study are outlined in Table 3. The cumulative event-free survival was found to be 98% by Kaplan-Meier method (Figure 2). Long term follow-up of the study would further confirm safety and efficacy.

## 4. Discussion

Long lesions are of particular concern, as these often require multiple stent implantations which lead to more extensive vascular injury and have been associated with increased risk of stent thrombosis and restenosis [15]. The distal vessel diameter, in case of long coronary lesion, is narrower making it unsuitable for coronary artery bypass grafting [7]. Many studies have demonstrated, by their evidences, that long length of coronary artery lesion is an independent key predictor of negative prognostic outcomes in terms of restenosis, after DES implementation [11, 16]. Kobayashi et al. [9] showed that longer stent length increases the risk of restenosis. Kastrati et al. [17] reported a relationship between lesion length and restenosis, the restenosis rates for lesions with lengths <15 mm and ≥15 mm being 28% and 37% (*P* < 0.001), respectively. Thus, long lesion disease continues to be a challenge for coronary intervention.

In this study, patient population had higher rates of hypertension (38.4%), 25.6% were smokers, multivessel disease (36%), and unstable angina (25.6%). The combination of these factors along with long lesions makes the patient population for this study atypically complex. In spite of such complications, cumulative MACE at 6-month follow-up was found to be only 2.0% which consisted of 2 cases of cardiac deaths and 3 cases of MI.

In earlier studies Indolimus has been successfully used in patients with considerably high-risk factors, in patients with acute myocardial infarction, and also in diabetics. These studies depict consistent efficacy and safety of Indolimus regardless of patient characteristics and complexities [18–22].

The SPIRIT PRIME trial [23], which included a smaller number of patients (*n* = 104) intervening 33 mm and 38 mm everolimus-eluting stents (EES), demonstrated its clinical outcomes at 1-year and 2-year follow-up in terms of MI, cardiac death, TLR, TLF, and ST. The results showed a higher incidence of MI 11 (10.6%) and TLR 3 (2.9%) at 1-year follow-up. This study showed no incidence of cardiac death or ST but in contrast there were greater risks associated when considering MI and TLR.

In a large, multicenter, real-world study of Chinese population with complex long lesion subset comprising 27.94% patients with diabetes and mean age of 59.59 years, XIENCE V demonstrated 0.0% death, 2.56% MI, 0.26% acute ST, and 2.56% TVF at 30 days [24]. Prevalence of adverse events in a study which compared resolute zotarolimus-eluting stent (ZES) with SES (LONG-DES IV) in long lesions was reported to be 12% and 14%, respectively, at 30-day follow-up and 14% and 16%, respectively, at 12-month clinical follow-up [8]. The proportion of males in LONG-DES IV study was comparable to our study. However, the number of diabetic patients was higher in present study as compared to both the arms of LONG-DES IV study, whereas, in LONG-DES-III study, the patients included were of older age than in present study and higher prevalence of hypertension and hyperlipidemia. However, the number of diabetic patients was less as compared to present study. The MACE at 1-month follow-up was 10.6% and 7.5% for EES and SES, respectively, and MACE at 12-month follow-up was 14.3% and 10.2%, respectively, for EES and SES [25]. The percentage of patients with previous history of PCI, MI, and CABG was also higher in the present study than the abovementioned studies. All previous studies show a variant rate of incidence of MACE, thus making the long lesion studies more convoluted. Therefore, investigating the efficacies of DES has important clinical implications for selecting the most effective therapy in high-risk lesion subsets. In summary, this study, held in “real-world” patients, presented favorable safety and efficacy profile of Indolimus in treatment of long lesions.

*Study Limitations*. The study holds a limitation of systematic diagnostic monitoring of the treated vessel not been carried out at follow-up.

## 5. Conclusions

The long (40 mm) Indolimus, a thin-strut, biodegradable polymer coated SES, demonstrated low MACE rate and was proven to be safe and effective treatment for long lesions in “real-world” patients.

## Figures and Tables

**Table 1:** Baseline demographic characteristics.

Characteristics	Indolimus SES *n* = 258 patients
Age (mean ± SD, yrs)	53.2 ± 11.0
Male, *n* (%)	192 (74.4%)
*Cardiovascular risk*	
Diabetes mellitus, *n* (%)	83 (32.2%)
Hypertension, *n* (%)	99 (38.4%)
Hypercholesterolemia, *n* (%)	21 (8.1%)
Smoker, *n* (%)	66 (25.6%)
Family history of CAD, *n* (%)	10 (3.9%)
Previous stroke, *n* (%)	4 (1.6%)
Previous MI, *n* (%)	25 (9.7%)
Previous PCI, *n* (%)	40 (15.5%)
Previous CABG, *n* (%)	7 (2.7%)
*Clinical presentation*	
Stable angina	18 (7.0%)
Unstable angina	66 (25.6%)

SES: sirolimus-eluting stent, CAD: coronary artery disease, MI: myocardial infarction, PCI: percutaneous coronary intervention, and CABG: coronary artery bypass grafting.

**Table 2:** Lesion and procedural characteristics.

Characteristics	Patients = 258/lesions = 278
Lesion location	
Left anterior descending, *n* (%)	100 (36.0%)
Right coronary artery, *n* (%)	141 (50.7%)
Left circumflex, *n* (%)	37 (13.3%)
Left main, *n* (%)	0 (0%)
ACC/AHA lesion classification	
C, *n* (%)	278 (100%)
Number of diseased vessels	
Single-vessel disease, *n* (%)	165 (64.0%)
Double-vessel disease, *n* (%)	79 (30.6%)
Triple-vessel disease, *n* (%)	14 (5.4%)
Total occlusion, *n* (%)	61 (21.9%)

Total number of stents	*N* = 280

Number of stents per patient (mean ± SD, mm)	1.1 ± 0.3
Number of stents per lesion (mean ± SD, mm)	1.0 ± 0.1
Average stent length (mean ± SD, mm)	40.0 ± 0.0
Average stent diameter (mean ± SD, mm)	3.1 ± 0.4

ACC/AHA: American College of Cardiology/American Heart Association.

**Table 3:** Cumulative clinical outcomes up to 6 months.

	In hospital (*n* = 258; 100%)	30-day follow-up (*n* = 258; 100%)	6-month follow-up (*n* = 256; 99.22%)
Death, *n* (%)	2 (0.8%)	2 (0.8%)	2 (0.8%)
Cardiac death, *n* (%)	2 (0.8%)	2 (0.8%)	2 (0.8%)
Noncardiac death, *n* (%)	0 (0%)	0 (0%)	0 (0%)
Myocardial infarction, *n* (%)	0 (0%)	0 (0%)	3 (1.2%)
Q-wave, *n* (%)	0 (0%)	0 (0%)	2 (0.8%)
Non-Q-wave, *n* (%)	0 (0%)	0 (0%)	1 (0.4%)
TLR, *n* (%)	0 (0%)	0 (0%)	0 (0%)
TVR, *n* (%)	0 (0%)	0 (0%)	0 (0%)
ST, *n* (%)	0 (0%)	0 (0%)	0 (0%)
Total	**2 (0.8%)**	**2 (0.8%)**	**5 (2.0%)**

TVR: target vessel revascularization; TLR: target lesion revascularization; ST: stent thrombosis.
